# Supplementary material for: The Effect of Portion Size Interventions on Energy Intake and Risk of Obesity in School-Aged Children: A Systematic Review and Meta-Analysis
Source: Nutrients. 2025 Sep 9;17(18):2911. doi: 10.3390/nu17182911 (PMC12472955; doi:10.3390/nu17182911)
Supplement: Supplementary file 1 [file nutrients-17-02911-s001.zip › nutrients-3814727-supplementary.pdf]

1. Medline (Ovid)

No	Search Terms
1	portion size/ or serving size/
2	portion size*.mp. [mp=title, book title, abstract, original title, name of substance word, subject heading word, floating sub-heading word, keyword heading word, organism supplementary concept word, protocol supplementary concept word, rare disease supplementary concept word, unique identifier, synonyms, population supplementary concept word, anatomy supplementary concept word]
3	serving size*.mp. [mp=title, book title, abstract, original title, name of substance word, subject heading word, floating sub-heading word, keyword heading word, organism supplementary concept word, protocol supplementary concept word, rare disease supplementary concept word, unique identifier, synonyms, population supplementary concept word, anatomy supplementary concept word]
4	1 or 2 or 3
5	energy intake/ or caloric restriction/
6	energy intake.mp. [mp=title, book title, abstract, original title, name of substance word, subject heading word, floating sub-heading word, keyword heading word, organism supplementary concept word, protocol supplementary concept word, rare disease supplementary concept word, unique identifier, synonyms, population supplementary concept word, anatomy supplementary concept word]
7	dietary intake.mp. [mp=title, book title, abstract, original title, name of substance word, subject heading word, floating sub-heading word, keyword heading word, organism supplementary concept word, protocol supplementary concept word, rare disease supplementary concept word, unique identifier, synonyms, population supplementary concept word, anatomy supplementary concept word]
8	calori* intake.mp. [mp=title, book title, abstract, original title, name of substance word, subject heading word, floating sub-heading word, keyword heading word, organism supplementary concept word, protocol supplementary concept word, rare disease supplementary concept word, unique identifier, synonyms, population supplementary concept word, anatomy supplementary concept word]
9	5 or 6 or 7 or 8

10	obesity/ or pediatric obesity/ or body weight/ or body weight changes/ or weight gain/ or overweight/
11	Body Mass Index/
12	body weight.mp. [mp=title, book title, abstract, original title, name of substance word, subject heading word, floating sub-heading word, keyword heading word, organism supplementary concept word, protocol supplementary concept word, rare disease supplementary concept word, unique identifier, synonyms, population supplementary concept word, anatomy supplementary concept word]
13	overweight.mp. [mp=title, book title, abstract, original title, name of substance word, subject heading word, floating sub-heading word, keyword heading word, organism supplementary concept word, protocol supplementary concept word, rare disease supplementary concept word, unique identifier, synonyms, population supplementary concept word, anatomy supplementary concept word]
14	body mass index.mp. [mp=title, book title, abstract, original title, name of substance word, subject heading word, floating sub-heading word, keyword heading word, organism supplementary concept word, protocol supplementary concept word, rare disease supplementary concept word, unique identifier, synonyms, population supplementary concept word, anatomy supplementary concept word]
15	BMI.mp. [mp=title, book title, abstract, original title, name of substance word, subject heading word, floating sub-heading word, keyword heading word, organism supplementary concept word, protocol supplementary concept word, rare disease supplementary concept word, unique identifier, synonyms, population supplementary concept word, anatomy supplementary concept word]
16	obesity.mp. [mp=title, book title, abstract, original title, name of substance word, subject heading word, floating sub-heading word, keyword heading word, organism supplementary concept word, protocol supplementary concept word, rare disease supplementary concept word, unique identifier, synonyms, population supplementary concept word, anatomy supplementary concept word]
17	10 or 11 or 12 or 13 or 14 or 15 or 16
18	Child/
19	child*.mp. [mp=title, book title, abstract, original title, name of substance word, subject heading word, floating sub-heading word, keyword heading word,

	organism supplementary concept word, protocol supplementary concept word, rare disease supplementary concept word, unique identifier, synonyms, population supplementary concept word, anatomy supplementary concept word]
20	p?ediatric*.mp. [mp=title, book title, abstract, original title, name of substance word, subject heading word, floating sub-heading word, keyword heading word, organism supplementary concept word, protocol supplementary concept word, rare disease supplementary concept word, unique identifier, synonyms, population supplementary concept word, anatomy supplementary concept word]
21	Pediatrics/
22	school age.mp. [mp=title, book title, abstract, original title, name of substance word, subject heading word, floating sub-heading word, keyword heading word, organism supplementary concept word, protocol supplementary concept word, rare disease supplementary concept word, unique identifier, synonyms, population supplementary concept word, anatomy supplementary concept word]
23	18 or 19 or 20 or 21 or 22
24	4 and 9 and 17 and 23

2. Embase (Ovid)

No	Search Terms
1	portion size/
2	serving size.mp. [mp=title, abstract, heading word, drug trade name, original title, device manufacturer, drug manufacturer, device trade name, keyword heading word, floating subheading word, candidate term word]
3	portion size.mp. [mp=title, abstract, heading word, drug trade name, original title, device manufacturer, drug manufacturer, device trade name, keyword heading word, floating subheading word, candidate term word]
4	1 or 2 or 3
5	caloric intake/
6	calori* intake.mp. [mp=title, abstract, heading word, drug trade name, original title, device manufacturer, drug manufacturer, device trade name, keyword heading word, floating subheading word, candidate term word]
7	energy intake.mp. [mp=title, abstract, heading word, drug trade name, original title, device manufacturer, drug manufacturer, device trade name, keyword heading word, floating subheading word, candidate term word]
8	dietary intake.mp. [mp=title, abstract, heading word, drug trade name, original title, device manufacturer, drug manufacturer, device trade name, keyword heading word, floating subheading word, candidate term word]
9	5 or 6 or 7 or 8
10	body weight/ or body weight change/
11	body weight gain/
12	obesity/
13	body mass/
14	body weight.mp. [mp=title, abstract, heading word, drug trade name, original title, device manufacturer, drug manufacturer, device trade name, keyword heading word, floating subheading word, candidate term word]
15	obesity.mp. [mp=title, abstract, heading word, drug trade name, original title, device manufacturer, drug manufacturer, device trade name, keyword heading word, floating subheading word, candidate term word]

16	overweight.mp. [mp=title, abstract, heading word, drug trade name, original title, device manufacturer, drug manufacturer, device trade name, keyword heading word, floating subheading word, candidate term word]
17	body mass index.mp. [mp=title, abstract, heading word, drug trade name, original title, device manufacturer, drug manufacturer, device trade name, keyword heading word, floating subheading word, candidate term word]
18	BMI.mp. [mp=title, abstract, heading word, drug trade name, original title, device manufacturer, drug manufacturer, device trade name, keyword heading word, floating subheading word, candidate term word]
19	childhood obesity/
20	10 or 11 or 12 or 13 or 14 or 15 or 16 or 17 or 18 or 19
21	child/
22	child*.mp. [mp=title, abstract, heading word, drug trade name, original title, device manufacturer, drug manufacturer, device trade name, keyword heading word, floating subheading word, candidate term word]
23	p?ediatric*.mp. [mp=title, abstract, heading word, drug trade name, original title, device manufacturer, drug manufacturer, device trade name, keyword heading word, floating subheading word, candidate term word]
24	pediatrics/
25	school age.mp. [mp=title, abstract, heading word, drug trade name, original title, device manufacturer, drug manufacturer, device trade name, keyword heading word, floating subheading word, candidate term word]
26	21 or 22 or 23 or 24 or 25
27	4 and 9 and 20 and 26

3. Cochrane

No	Search Terms
#1	MeSH descriptor: [Portion Size] this term only
#2	MeSH descriptor: [Serving Size] this term only
#3	"portion size" or "portion sizes"
#4	#1 or #2 or #3
#5	MeSH descriptor: [Energy Intake] explode all trees
#6	"energy intake"
#7	"dietary intake"
#8	"calorie intake" or "caloric intake" or "calories intake"
#9	#5 or #6 or #7 or #8
#10	MeSH descriptor: [Body Weight] this term only
#11	MeSH descriptor: [Body Weight Changes] this term only
#12	MeSH descriptor: [Weight Gain] this term only
#13	MeSH descriptor: [Overweight] this term only
#14	MeSH descriptor: [Obesity] this term only
#15	MeSH descriptor: [Pediatric Obesity] this term only
#16	"body weight"
#17	overweight
#18	"body mass index"
#19	BMI
#20	obesity
#21	#10 or #11 or #12 or #13 or #14 or #15 or #16 or #17 or #18 or #19 or #20
#22	MeSH descriptor: [Child] this term only
#23	MeSH descriptor: [Pediatrics] this term only
#24	child*
#25	pediatric*
#26	paediatric*
#27	"school age"
#28	#22 or #23 or #24 or #25 or #26 or #27
#29	#4 and #9 and #21 and #28
